# Potential Utility of *Bacillus amyloliquefaciens* SFB-1 as a Biocontrol Agent for Sweetpotato Black Rot Caused by *Ceratocystis fimbriata*

**DOI:** 10.3390/genes15121540

**Published:** 2024-11-28

**Authors:** Fangyuan Gao, Xiaosi Zhou, Dongjing Yang, Jingwei Chen, Veronica Tshegofatso Kgosi, Chengling Zhang, Jukui Ma, Wei Tang, Zhao Liang, Houjun Sun

**Affiliations:** 1 Key Laboratory of Biology and Genetic Improvement of Sweetpotato, Xuzhou Institute of Agricultural Sciences in Jiangsu Xuhuai District, Ministry of Agriculture, Xuzhou 221131, China; 2 Jiangsu Coastal Area Institute of Agricultural Sciences, Yancheng 224002, China; 3 Department of Plant Pathology, College of Plant Protection, Nanjing Agricultural University, Nanjing 210095, China

**Keywords:** sweetpotato, *Ceratocystis fimbriata*, biocontrol, *Bacillus amyloliquefaciens*, transcriptome

## Abstract

**Background/Objectives**: Sweetpotato black rot, caused by *Ceratocystis fimbriata*, is a severe fungal disease in sweetpotato production. Biological control strategies represent a promising, environmentally sustainable approach to managing this disease. This study investigates the biocontrol potential of *Bacillus amyloliquefaciens* SFB-1 against *C. fimbriata*. **Methods**: The antagonistic activities of strain SFB-1 on *C. fimbriata* were assessed through *in vitro* assays, including evaluations of mycelial inhibition, spore germination, and mycelial morphology. Pathogenicity assays on harvested sweetpotato roots assessed lesion diameter and depth. A transcriptomic analysis of *C. fimbriata* exposed to strain SFB-1 was performed to explore the underlying antifungal mechanism of SFB-1 on *C. fimbriata*. The qRT-PCR was employed to validate the RNA-seq results. **Results**: *In vitro* assays demonstrated that strain SFB-1 inhibited *C. fimbriata* mycelial growth by up to 81.01%, caused mycelial swelling, and completely suppressed spore germination at 10^8^ CFU/mL. The cell-free supernatant of strain SFB-1 also suppressed *C. fimbriata* growth. Pathogenicity assays revealed that strain SFB-1 treatments reduced lesion diameter and depth on harvested sweetpotato roots by over 50% compared to untreated controls. Transcriptomic analysis of *C. fimbriata* treated with strain SFB-1 identified 1164 differentially expressed genes, with significant alterations in genes associated with cell wall integrity, cell membrane stability, spore germination, detoxification, and antioxidant responses. The qRT-PCR validation of 16 genes confirmed the consistency with the RNA-seq results. **Conclusions**: *B. amyloliquefaciens* SFB-1 demonstrates significant biocontrol efficacy against *C. fimbriata* through multiple mechanisms, positioning it as a promising solution for the sustainable management of sweetpotato black rot.

## 1. Introduction

Sweetpotato (*Ipomoea batatas* (L.) Lam.), a globally significant crop, is extensively cultivated for its dual utility as a nutritional food source and an essential industrial raw material [1]. However, sweetpotato often face the threat of pathogens during the growth and storage stage. Sweetpotato black rot, a fungal disease caused by *C*. *fimbriata* is one of the most devastating fungal diseases in the production of sweetpotato [2]. It has been found in nearly all tropical and subtropical production areas worldwide, leading to yield losses of 10–20%, and occasionally up to 20–50% [3]. When sweetpotato black rot occurs, lesions appear on the surface of sweetpotatoes that are typically round or oval, brown, and slightly depressed, often accompanied by a bitter taste [4,5]. Meanwhile, infected sweetpotatoes can produce toxic substances, such as ipomeamarone and derivatives, which are harmful to humans and livestock [6].

Various strategies have been explored to manage sweetpotato black rot, including physical methods, agricultural practices, essential oils, and chemical fungicides [3]. Physical methods (curing and improved storage, etc.) are underutilized due to cost and inconsistent efficacy. Essential oils have demonstrated effectiveness against *C. fimbriata* [7,8], but standardized application protocols are lacking [9]. Agricultural measures require significant labor and time, reducing profitability. Although disease-resistant breeding is an effective control strategy, no variety has exhibited complete resistance to sweetpotato black rot [10]. Traditional chemical fungicides, such as carbendazim and difenoconazole, have been used to manage pathogenic fungi [11,12]. However, their prolonged and widespread use has resulted in increased resistance and adverse environmental effects [13]. Therefore, there is an urgent need for cost-effective and environmentally sustainable strategies to control sweetpotato black rot.

Biological control agents offer a safe and promising approach for managing agricultural diseases [14], due to their adaptability to the environment and ability to produce antifungal substances [15,16]. In recent years, *Bacillus*, including *B. subtilis* [17,18,19,20,21], *B. altitudinis* [22,23,24,25], *B. velezensis* [26,27,28,29], and *B. amyloliquefaciens* [30,31,32,33,34], have been utilized to control agricultural diseases. For instance, a treatment with *B. subtilis* HSY21 can significantly reduce soybean root rot by suppressing the expression of genes related to the pathogenicity of *Fusarium oxysporum* [21]. *B. amyloliquefaciens* FG14 significantly inhibits mycelial growth, spore production, and spore germination of *Ilyonectria robusta*, which causes ginseng rusty root rot, with an inhibitory rate of more than 90%. The sterile fermentation of strain FG14 also had an obvious inhibitory effect on the pathogen [16]. The cell-free supernatant (CFS) of *B. velezensis* S161 effectively inhibited *Penicillium digitatum* mycelial growth, spore germination, and germ tube elongation, which was associated with the downregulation of genes involved in spore germination, growth, reproduction, and virulence [35]. *Bacillus* species have been shown to be effective biocontrol agents. They are therefore promising tools for protecting crops from post-harvest fungal diseases.

*B. amyloliquefaciens* is currently being used to control black rot in sweetpotato. For example, *B. amyloliquefaciens* XZ-1 isolated from the sweetpotato rhizosphere could significantly inhibit mycelial growth and spore germination of *C. fimbriata*, and the cell-free supernatant (CFS) was able to significantly reduce the volume of the spot, with a control effect of 65.02% [36]. *B. amyloliquefaciens* YTB1407 isolated from the root of *Panax quinquefolium* L had an obvious antifungal effect on mycelia of *C. fimbriata in vitro* and *in vivo* [37]. The pretreatment of YTB1407 suspensions could increase the salicylic acid content and upregulate the expression of salicylic acid pathway-related genes (*NPR1*, *PR1*). Although *B. amyloliquefaciens* has been explored for controlling sweetpotato black rot, its effectiveness and the mechanisms involved are not yet fully understood.

The strain SFB-1 was isolated from the rhizosphere soil of healthy chrysanthemum plants cultivated in Yan’cheng city, Jiangsu Province, and was identified as *B*. *amyloliquefaciens*. Recent studies have demonstrated that strain SFB-1 effectively inhibits the mycelial growth of several plant pathogenic fungi *in vitro* (the data are currently being published). Consequently, the primary objectives of this study are to (1) test the antifungal activities of strain SFB-1 against *C. fimbriata* growth *in vitro*; (2) evaluate the biocontrol efficacy of strain SFB-1 against *C. fimbriata* during the postharvest storage; and (3) investigate the potential antifungal mechanism of *C. fimbriata* in response to strain SFB-1 by transcriptome analysis. The results of this study may provide valuable insights into the biological control of sweetpotato black rot after the harvest.

## 2. Materials and Methods

### 2.1. C. fimbriata Strain, Bacterial Strain, and Culture Conditions

The *C. fimbriata* strain used in this study was previously isolated from diseased sweetpotato tubers, identified by ITS sequencing, and stored at the Jiangsu Xuzhou Sweetpotato Research Center. It was cultured on potato dextrose agar (PDA) medium (200 g/L potato, 20 g/L dextrose, and 16 g/L agar powder). *B. amyloliquefaciens* SFB-1, isolated from the rhizosphere soil of healthy chrysanthemum plants, was cultured on Luria Bertani (LB) agar (1% Tryptone, 0.5% yeast extract, 1% NaCl, and 1.5% agar) at 37 °C. [*Ipomoea batatas* (L.) Lam. cv. Pu shu 32] was used in this study.

### 2.2. In Vitro Antagonism Assays

After 7 days of activation culture, fresh mycelial plugs (5 mm in diameter) were taken from the margins of the *C. fimbriata* colony using a hole punch. Two pieces of sterilized paper containing SFB-1 suspension (10^7^ CFU/mL) were placed symmetrically at a distance of 2.5 cm from the center of mycelial plugs, and sterilized paper with 2 µL sterilized water as the control. The average colony diameters were recorded after 4, 7, 10, 13, and 16 days of incubation at 28 °C in a growth chamber. The growth inhibition rate was calculated using the following formula: Inhibition rate (%) = (colony diameter of the control − colony diameter of the treatment)/(colony diameter of the control) × 100.

### 2.3. Effects of Strain SFB-1 Treatment on C. fimbriata Mycelial Morphology and Vegetative Growth

To analyze the effects of SFB-1 on *C. fimbriata* mycelial morphology, the fungus dish was inoculated into 50 mL PDA liquid medium. For the treatment group, 2 µL of SFB-1 bacterial suspension (1 × 10^7^ CFU/mL) was added, and 2 µL of sterile water was added as the control group. The growth of mycelia was incubated in the dark at 28 °C for 3, 5, and 7 days. The mycelial morphology was observed under a microscope at each time point. Each treatment was conducted with three biological replicates and repeated three times.

### 2.4. Effects of Strain SFB-1 on C. fimbriata Spore Germination

The previous method [38] was used to analyze the germination of *C. fimbriata* spores. *C. fimbriata* was cultured on PDA medium at 28 °C for 7 days. The spores were washed with sterile water, and the concentration was adjusted to 1 × 10^6^ spores/mL for subsequent experiments. Each milliliter of spore suspension was placed in a separate 2 mL centrifuge tube. A 1 mL sample of liquid PDA was used as the control. Different concentrations of SFB-1 (1 ×10^3^, 1 × 10^4^, 1 × 10^5^, 1 × 10^6^, 1 × 10^7^, 1 × 10^8^ CFU/mL) were added to each centrifuge tube and mixed evenly. The treated spores were incubated at 28 °C for 16 h. The spore germination was observed and recorded under a microscope, with at least 100 spores counted per sample. The germination rate and germination inhibition rate were calculated using the following formulas: germination rate (%) = total number of germinated spores/total number of observed spores × 100; germination inhibition rate (%) = (total observed spores − number of germinated spores)/(total observed spores) × 100. Spore germination and inhibition were evaluated with no fewer than three replicates per sample.

### 2.5. Effects of the Cell-Free Supernatant (CFS) and Volatile Organic Compounds (VOCs) from Strain SFB-1 on Mycelial Growth of C. fimbriata

The inhibitory effects of volatile organic compounds (VOCs) produced by strain SFB-1 on *C. fimbriata* were evaluated using a modified version of the method described by Yousefvand [39]. Divided Petri dishes (90 mm diameter) were used, with one compartment inoculated with a *C. fimbriata* mycelial plug, and the other inoculated with strain SFB-1. The control group consisted of *C. fimbriata* cultured independently. All plates were incubated at 28 °C and colony diameters were measured on day 7. The effect of the CFS of strain SFB-1 on mycelial growth was detected following the method outlined by Liu et al. [38]. Each experiment included three biological replicates and was repeated three times.

### 2.6. The Biocontrol Efficacy of SFB-1 Strain Against Sweetpotato Black Rot

Healthy sweetpotato tuberous roots were stored at Jiangsu Xuzhou Sweetpotato Research Center. They were washed clean with sterile water and surface-sterilized using 75% ethanol. After natural drying at room temperature, a sterilized hole punch was used to make five cavities (0.2 cm in diameter, 0.5 cm in depth) in each sweetpotato tuber. For the control, 20 µL of sterile water was added to the cavities. In the treatment group, 20 uL strain SFB-1 suspension (OD 600 = 0.6) was added to the cavities for one day. A 2 µL suspension of *C. fimbriata* spores (1 × 10^6^ spores/mL) was inoculated into the cavities. All samples were then incubated at 28 °C for 10 days. Disease severity was assessed by measuring the diameter and depth of the infected wounds. Three biological replicates were used for each treatment.

### 2.7. Transcriptome Analysis of the C. fimbriata Response to SFB-1

The mycelia treated with strain SFB-1 were obtained following a modified method as described by Sun [40]. Sterilized yellow pipettes were used to collect mycelia from the edges of colonies cultured on PDA medium with SFB-1 for 5 days. *C. fimbriata* mycelia grown on PDA without SFB-1 served as the control. Mycelia from 20 colonies were pooled to form one replicate, and three replicates were immediately frozen in liquid nitrogen for RNA extraction.

Total RNA was extracted using the TIANGEN Polysaccharide and Polyphenol Plant Total RNA Extraction Kit according to the manufacturer’s instructions (Tiangen Bio-Tech (Beijing) Co., Ltd., Beijing, China, Catalogue No. DP441). The quality and integrity of the RNA samples, library construction, and sequencing using an Illumina HiSeq 2500 platform were ensured by using GENE DENOVE. Low-quality reads were removed to ensure high-quality clean data, which were subsequently mapped to the *C. fimbriata* reference genome (GCA_000389695.3) using HISAT2. The fragments per kilobase per million (FPKM) values were used to estimate the expression patterns of genes. FDR (|log_2_(fold-change)| ≥ 1, *p* < 0.05) was used as the standard for the screening of differential gene expression. The enrichment analysis of Gene Ontology (GO) and Kyoto Encyclopedia of Genes and Genomes (KEGG) were performed using the free online Omicsmart (www.majorbio.com accessed on 12 June 2024).

### 2.8. qRT-PCR Analysis

Quantitative real-time reverse transcription PCR (qRT-PCR) was carried out using the same RNA samples extracted as described above. The reactions were performed with a ChamQ Universal SYBR Green qPCR Master Mixt (Vazyme, Nanjing, China). The qRT-PCR system and gene expression quantification were performed following the protocol outlined by Gong, with gene expression levels normalized to the reference gene *actin-related protein 3* (*arp3*) and calculated using the 2^−ΔΔCt^ method [41]. All reactions were conducted in triplicate, and the primers are listed in Supplementary Table S1.

### 2.9. Statistical Analysis

Statistical analyses were conducted using GraphPad Prism 8 software (GraphPad Software, San Diego, CA, USA). Data are presented as means ± standard deviation (SD) or standard error (SE), as indicated. For comparisons between two independent groups, an unpaired *t*-test was used, while one-way analysis of variance (ANOVA) followed by Tukey’s multiple-range test was applied for multiple group comparisons. Statistical significance was considered at *p* < 0.05. Statistical differences between groups were further indicated using different lowercase letters to denote significant differences. All experiments were conducted in triplicate, and data from independent experiments were pooled for analysis. Graphical representations were generated using GraphPad Prism 8 software. Statistical significance is indicated as follows: *** *p* < 0.001.

## 3. Results

### 3.1. Antagonistic Activity of Strain SFB-1 Against C. fimbriata In Vitro

**Figure 1:**
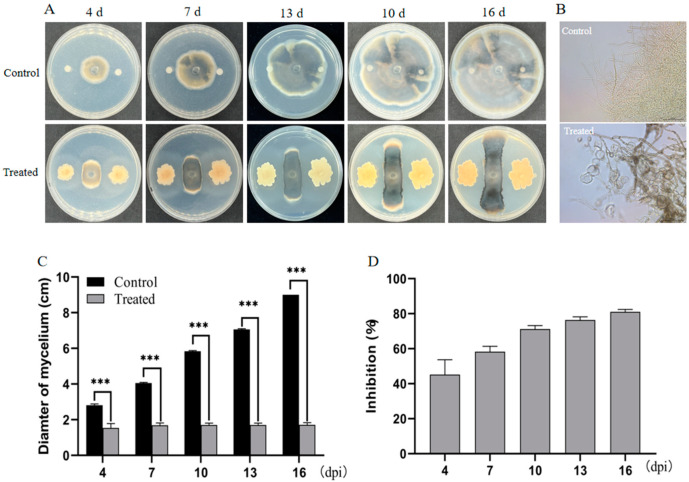
Antagonistic activity of strain SFB-1 against *C. fimbriata* *in vitro*. (**A**) Dual-culture assay of *C. fimbriata* and strain SFB-1 on PDA medium. (**B**) Effect of strain SFB-1 on *C. fimbriata* mycelia. *C. fimbriata* mycelia were treated with strain SFB-1, and without strain SFB-1, and micrographs were obtained. (**C**) Diameter of *C. fimbriata* mycelial growth and inhibition of mycelial growth. (**D**) Visual representation of inhibition rate of strain SFB-1 on mycelial diameter. Significance levels: *** *p* < 0.001.

A plate antagonism test was conducted to investigate the effects of SFB-1 on the growth of *C. fimbriata*. Mycelia could grow normally without SFB-1 treatment. In the group with SFB-1, mycelial growth was significantly inhibited (Figure 1A,C). Additionally, SFB-1 induced swelling in many of the mycelia (Figure 1B). After 3, 7, 10, 13, and 16 days of mycelial culture, the inhibition rates of mycelial growth were 45.25%, 58.27%, 71.24%, 76.35%, and 81.01%, respectively (Figure 1D). These results indicated that SFB-1 exhibited potent antifungal activity to strongly inhibit mycelial growth.

### 3.2. Strain SFB-1 Markedly Changed C. fimbriata Mycelial Morphology

**Figure 2:**
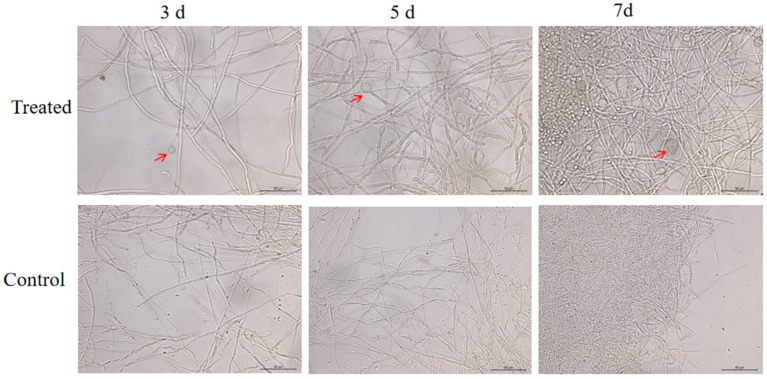
Effect of *B*. *amyloliquefaciens* SFB-1 on the morphology of *C. fimbriata* mycelia at different times. Mycelia of *C. fimbriata* were treated with sterile water and strain SFB-1 at the concentration of 1 × 10^7^ CFU/mL. Arrows indicate mycelial swelling. Scale bar indicates 50 μm.

To further investigate the effect of SFB-1 on *C. fimbriata* mycelia, we observed the control and the SFB-1-treated mycelia at different times. At 3 d, 5 d, and 7 d, mycelial morphology was observed under the microscope. In SFB-1-treated mycelia, the frequency of mycelial swelling increased over time, while the mycelia treated without SFB-1 were normal, with smooth surfaces and normal branches (Figure 2).

### 3.3. Strain SFB-1 Significantly Suppressed Spore Germination

**Figure 3:**
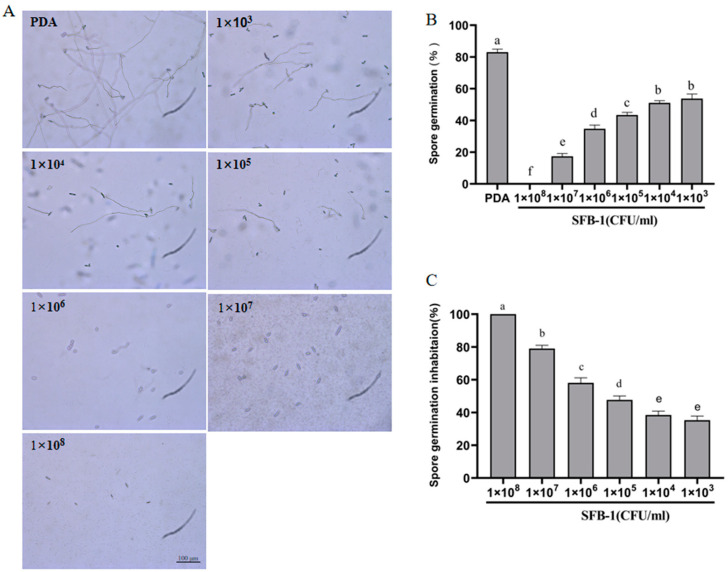
Effects of *B*. *amyloliquefaciens* SFB-1 at various concentrations on spore germination of *C. fimbriata* at 16 hpi. (**A**) Effects of strain SFB-1 on spore germination rates. (**B**,**C**) Spore germination rates and inhibition rates of *C. fimbriata* treated with strain SFB-1 at different concentrations (1 × 10^3^, 1 × 10^4^, 1 × 10^5^, 1 × 10^6^, 1 × 10^7^, 1 × 10^8^ CFU/mL) at 16 hpi. Untreated spores served as the control. Means with different letters for each strain SFB-1 concentration denote significant differences (*p* < 0.001).

The *C. fimbriata* spore suspensions were incubated with different concentrations of strain SFB-1 for 16 h. Microscopy revealed that the spore germination rate of the SFB-1-treated group was significantly lower than that of the control group (Figure 3A). The spore germination rates for spores treated with different concentrations of SFB-1 (1 × 10^3^, 1 × 10^4^, 1 × 10^5^, 1 × 10^6^, 1 × 10^7^, 1 × 10^8^ CFU/mL) were 53.74%, 51.09%, 43.39%, 34.73%, 17.38%, and 0%, respectively (Figure 3B). When the concentration of spores treated with SFB-1 bacterial solution reached 1 × 10^8^ (CFU/mL), the inhibition rate of spore germination was 100% (Figure 3C). The results demonstrate that strain SFB-1 effectively inhibited germination of *C. fimbriata* spores.

### 3.4. Strain SFB-1 CFS Has Antifungal Activity

**Figure 4:**
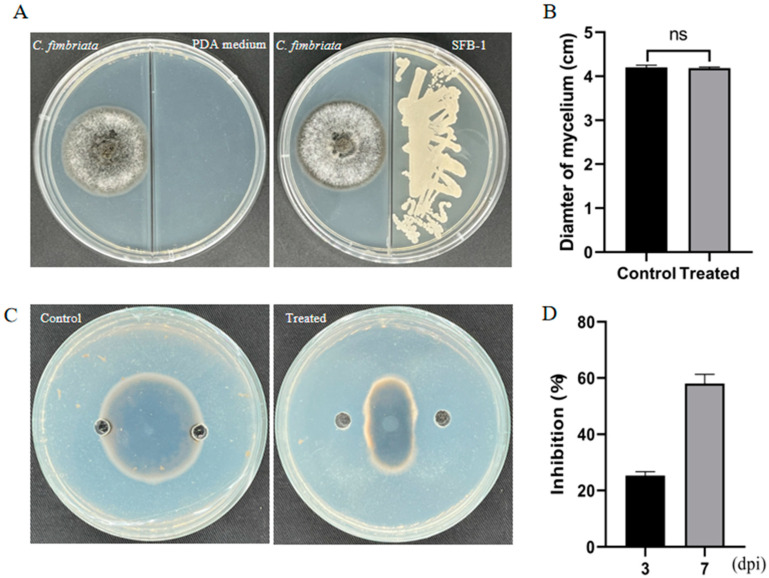
Antagonism capacity of strain SFB-1 VOCs and CFS against *C. fimbriata*. (**A**) The growth inhibition results of strain SFB-1 VOCs on *C. fimbriata* on PDA plate. (**B**) The mycelial diameters of the control groups and treatment groups on day 7. “ns” represented no significant difference. (**C**) The growth inhibition results of strain SFB-1 CFS to *C. fimbriata* on PDA plate. (**D**) Inhibition rate of the CFS on mycelia on the third and seventh days.

The above results indicated that SFB-1 was capable of inhibiting the mycelial growth of *C. fimbriata*. To further explore the underlying inhibition mechanism, we assessed the antifungal activities of its CFS and VOCs. The results revealed that CFS reduced mycelial growth on PDA medium by 58.05% on day 7 (Figure 4C,D), while VOCs produced by SFB-1 showed no detectable inhibitory effect on mycelial growth (Figure 4A,B). These results indicated that SFB-1 primarily inhibited mycelial growth through the secretion of CFS.

### 3.5. Strain SFB-1 Inhibited Black Rot Disease of Sweetpotato

**Figure 5:**
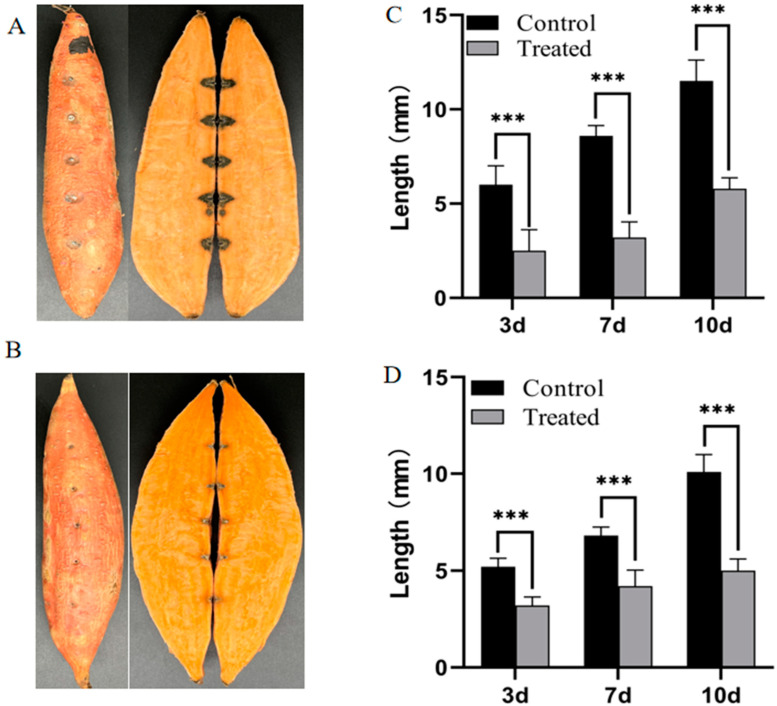
Biocontrol efficacy of strain SFB-1 against *C. fimbriata* on sweetpotato storage roots. (**A**,**B**) The symptoms of black rot disease on sweetpotato in control samples and in strain SFB-1-treated samples on day 10. (**C**,**D**) Statistics of disease spot diameter and depth. Significance levels: *** *p* < 0.001.

To estimate the potential utility of SFB-1 for detecting and controlling *C. fimbriata*, the strain SFB-1 and *C. fimbriata* spores were inoculated into sweetpotato storage roots. With the increase in storage time, lesion diameter and depth grew in both the control and SFB-1 treatment groups. After 10 days of inoculation, the sweetpotato storage roots exhibited typical lesions at the inoculation site (Figure 5A,B). The mean lesion diameter and depth of sweetpotato without SFB-1 were 12 mm and 10 mm, respectively, and those for sweetpotato with SFB-1 treatment were 5 mm and 5 mm (Figure 5C,D). The average lesion diameter and depth of SFB-1-treated samples were significantly lower than those of sweetpotato without SFB-1, indicating that SFB-1 could effectively inhibit the spread of sweetpotato black rot.

### 3.6. Transcriptomic Analysis C. fimbriata to Strain SFB-1

**Figure 6:**
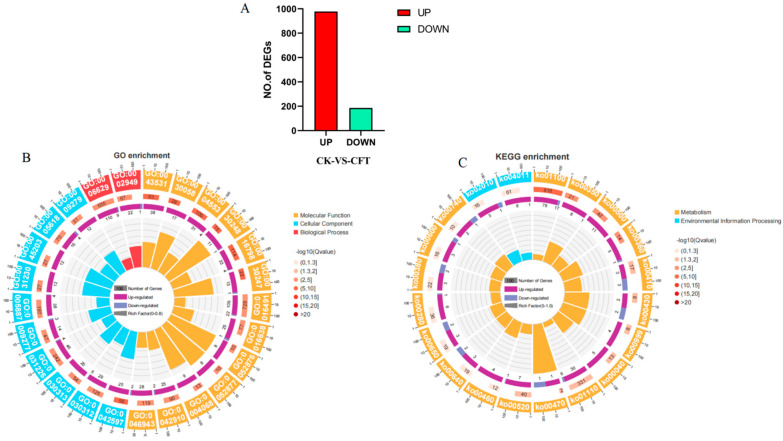
DEGs analysis of *C. fimbriata* after SFB-1 treatment. (**A**) Number of upregulated and downregulated DEGs after SFB-1 treatment. (**B**) Enriched GO terms significantly enriched (*p* < 0.05) with DEGs. (**C**) KEGG pathways significantly enriched (*p* < 0.05) with DEGs.

To further investigate the mechanism by which strain SFB-1 inhibits *C. fimbriata*, we performed transcriptome sequencing on *C. fimbriata* treated with strain SFB-1. By filtering the raw reads, 35.51 Gb of clean sequencing data were obtained (Supplementary Table S2). The samples displayed GC content above 50%, with Q20 and Q30 values exceeding 96.50% and 90.77%, respectively, confirming their reliability for bioinformatic analyses (Supplementary Table S2) and ensuring high data quality for further analysis. Differential expression analysis identified 1164 DEGs, of which 978 (84.02%) were upregulated and 186 (15.98%) were downregulated (Figure 6A). GO enrichment analysis revealed significant associations with cellular processes (GO:0009987), metabolic processes (GO:0008152), and biological regulation (GO:0065007) as the predominant biological processes (Figure 6B, Supplementary Table S3). Enriched cellular components included cellular anatomical entities (GO:0110165) and protein-containing complexes (GO:0032991). The primary molecular functions linked to upregulated DEGs were binding (GO:0005488) and catalytic activity (GO:0003824) (Figure 6B, Supplementary Table S3). KEGG pathway analysis indicated that metabolic pathways (ko01100) and the biosynthesis of secondary metabolites (ko01110) were the most prominently enriched pathways (Figure 6C, Supplementary Table S4). Similarly, the downregulated DEGs were primarily associated with metabolic pathways (ko01100) and biosynthesis of secondary metabolites (ko01110), followed by β-alanine metabolism (ko00410), carbon metabolism (ko01200), propanoate metabolism (ko00640), and tryptophan metabolism (ko00380) (Figure 6C, Supplementary Table S4).

**Figure 7:**
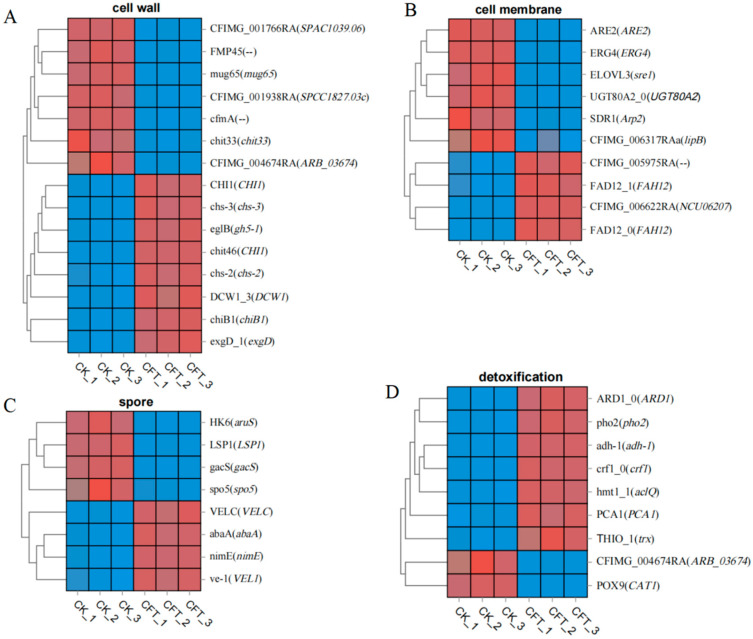
Heatmaps showing the relative expression of selected DEGs in *C. fimbriata* following SFB-1 treatment. The log_2_ (fold-change) was colored and standardized. Colors indicate DEGs in strain SFB-1-treated *C. fimbriata* mycelia versus the control. Red, upregulated; blue, downregulated. (**A**–**D**) DEGs involved in (**A**) cell wall, (**B**) cell membrane, (**C**) spore development, and (**D**) detoxification.

Through GO and KEGG analyses, we identified DEGs involved in cell wall integrity, the cell membrane structure, spore germination, detoxification, and antioxidant activities (Figure 7). Overall, the number of upregulated and downregulated genes was comparable. However, genes involved in cell wall integrity, cell membrane structure, and spore formation were predominantly downregulated, suggesting that strain SFB-1 may disrupt these essential physiological functions in *C. fimbriata* by suppressing the expression of related genes. Conversely, detoxification-related genes were generally upregulated, indicating that *C. fimbriata* might activate detoxification mechanisms to counteract the external stress induced by SFB-1 treatment.

### 3.7. Validation of RNA-Seq Sequencing

**Figure 8:**
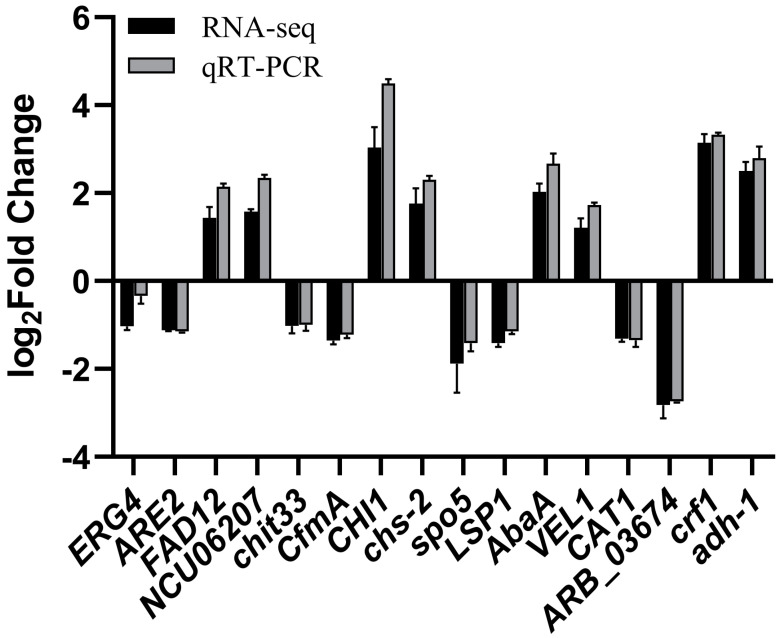
Relative expression levels of 16 DEGs involved in cell membrane integrity, cell wall biosynthesis, spore development, and detoxification processes assessed using RNA-seq and qRT-PCR.

Sixteen DEGs associated with cell membrane structure, cell wall integrity, spore germination, detoxification, and antioxidant activity were randomly chosen for verification via qRT-PCR analysis. The results were consistent with the trend of transcription profiles from RNA-seq data (Figure 8).

## 4. Discussion

In this study, the *B*. *amyloliquefaciens* strain SFB-1 was shown to effectively inhibit the mycelial growth, and spore germination of *C. fimbriata* (Figure 1 and Figure 3) and reduce the size and depth of sweet potato black rot (Figure 5). In addition, the CFS (Figure 4C) produced by strain SFB-1 had antifungal activities, which is consistent with the findings of previous research on *Bacillus* species [35,42]. A transcriptomic analysis was conducted to explore the potential mechanism of SFB-1 against *C. fimbriata*.

The integrity of the fungal cell wall is crucial for pathogen survival and adaptability, especially under biotic stress [2]. Chitin and glucan are two major components of the fungal cell wall. Our transcriptomic analysis of *C. fimbriata* exposed to *B. amyloliquefaciens* SFB-1 revealed significant differential expression of genes related to cell wall biosynthesis and stability. Key genes, including *CHI1*, *chs-2*, *chs-3*, *chiB1*, *gh5-1*, and *exgD*, were upregulated 3.04-fold, 1.76-fold, 1.72-fold, 1.64-fold, 2.88-fold, and 1.06-fold after SFB-1 treatment (Figure 7A). These genes are crucial for chitin and glucan metabolism, integral components of the fungal cell wall, suggesting an attempt by *C. fimbriata* to strengthen its cell wall against the stress induced by SFB-1. Conversely, *ARB_03674*, *SPAC1039.06*, *chit33*, *mug65*, and *SPCC1827.03c* were downregulated 2.82-fold, 1.24-fold, 1.02-fold,1.09-fold, and 2.35-fold (Figure 7A). These genes play roles in cell wall remodeling and stress response. The observed swelling in SFB-1-treated mycelia may be due to a disruption in cell wall synthesis, similar to the effects seen in chitin synthase mutants [43]. The transcriptomic changes observed in *C. fimbriata* in response to *B. amyloliquefaciens* SFB-1 treatment highlight a dual strategy: upregulation of genes to bolster cell wall synthesis and downregulation of genes involved in cell wall degradation. This coordinated response likely aims to enhance cell wall strength and mitigate damage.

Lipids are the main component of cellular membranes and affect the shape and structure of the membrane [44]. In the present study, *FAD12* and *NCU06207* involved in fatty acid desaturation and biosynthesis were upregulated 1.58-fold and 2.12-fold, respectively, after SFB-1 treatment (Figure 7B). However, genes *lipB* and *Arp2*, related to fatty acid biosynthetic process, were downregulated 1.56-fold and 1.12-fold, respectively (Figure 7B). Ergosterol (ERG) is a critical sterol in fungal cell membranes, primarily responsible for maintaining membrane stability and fluidity [45,46]. The *ERG4* encodes an enzyme responsible for catalyzing the terminal step of the ergosterol biosynthesis pathway [47]. The sterol-acyl transferase encoded by the *ARE2* gene positively regulates the accumulation of ergosterol in the Saccharomyces cerevisiae [48,49]. In *Xanthophyllomyces dendrorhous*, *Sre1*, a sterol regulatory element-binding protein, directly regulates genes involved in ergosterol biosynthesis, highlighting its crucial role in coordinating sterol homeostasis and carotenoid production [50]. In an Arabidopsis mutant, the biosynthesis of steryl glycosides is severely impaired due to the inactivation of the two sterol glucosyltransferases, *UGT80A2* and *UGT80B1* [51]. In the current study, the downregulated expression of *ERG4*, *ARE2*, *UGT80A2*, and *Sre1* (1.03-fold, 1.12-fold, 1.14-fold, and 1.05-fold, respectively) (Figure 7B), which are involved in ergosterol biosynthesis, may alter the membrane components as well as membrane trafficking and signal transduction. This means that *B. amyloliquefaciens* SFB-1 is likely to cause lesions on the cell membrane by altering membrane component-related genes, potentially affecting membrane permeability, as observed in previous studies of *Pseudomonas chlororaphis* subsp. *aureofaciens* SPS-41 volatile treatment [52].

Fungal spores, which exhibit considerable variability and high stress resistance, are essential for the dispersal of pathogenic fungi to new habitats and play a critical role in propagation and infection [53]. The transcriptional profiling revealed that four genes, *AbaA*, *VELC*, *VEL1*, and *fluG*, were significantly upregulated 1.99-fold, 4.23-fold, and 3.97-fold, respectively (Figure 7C). These genes are critical in the context of fungal sporulation and germination. For example, in *Metarhizium robertsii*, the transcription factor *AbaA* is essential for spore production, as it positively regulates conidiation and blastospore separation by modulating the expression of genes such as *Mr-veA* and *Mr*-*wetA* [54]. In *Aspergillus nidulans*, the *VelC* gene regulates spore production by modulating the expression of key genes such as *brlA*, *abaA*, *wetA*, and *vosA*, which are involved in asexual sporulation and sexual development, thereby balancing conidia formation and cleistothecia production [55]. In *Verticillium dahliae*, the *Vel1* gene is essential for spore production and conidiation, playing a pivotal role in the initial plant root colonization and propagation in planta [56]. Conversely, genes encoding Sporulation-specific protein 5 (*spo5*), putative histidine kinase 6 (*aruS*), Sphingolipid long chain base-responsive protein PIL1 (*LSP1*), and sensor protein GacS (*gacS*) were downregulated 7.61-fold, 1.64-fold, 5.95-fold, and 5.03-fold, respectively (Figure 7C). The downregulation of these genes was associated with spore development, suggesting a potential disruption in vital cellular processes necessary for spore germination and development. Specifically, the gene *spo5* is known to play a role in the transition from spore dormancy to germination [57], indicating that SFB-1 could hinder the transition to active growth in *C. fimbriata*.

Plant pathogenic fungi have developed various mechanisms to improve their tolerance to diverse stresses [58,59]. Examples include the regulation of detoxification and antioxidant-related genes [60] to minimize damages [61]. The thioredoxin (Trx) system, comprising NADPH, thioredoxin reductase (TrxR), and thioredoxin, is a pivotal antioxidant mechanism that mitigates oxidative stress by regulating the dithiol/disulfide balance through disulfide reductase activity. It plays a vital role in immune responses, viral infections, and cell death, primarily through its interaction with thioredoxin-interacting protein [62]. Moreover, alcohol dehydrogenase is associated with the detoxification of alcohols and aldehydes, contributing to cellular protection against toxic metabolites [63]. Consistent with preceding studies, we found that the genes encoding thioredoxin-like protein (*trx*) and alcohol dehydrogenase 1 (*adh-1*) had upregulated 1.17-fold and 2.59-fold, respectively, in *C. fimbriata* exposed to SFB-1. The gene *ARD1*, which was also upregulated 1.34-fold (Figure 7D), is involved in N-terminal acetylation, a modification critical for protein stability and function [64]. Its increased expression may be indicative of a cellular response to maintain protein integrity under stress conditions. Genes encoding P-type cation-transporting ATPase (*PCA1*), heavy metal tolerance protein (*aclQ*), 4-nitrophenylphosphatase (pho2), and putative glycosidase crf1 (*crf1*) were upregulated 1.34-fold, 1.23-fold, 1.81-fold, and 3.22-fold, respectively, after SFB-1 treatment. Gene ontology (GO) enrichment analysis showed that these genes were mainly involved in detoxification and the detoxification of inorganic compounds, highlighting a broad-spectrum detoxification response. These results indicated that *C. fimbriata* can upregulate antioxidant and detoxification-related genes to improve the detoxification efficiency under the persistent biological stress of *B. amyloliquefaciens* SFB-1. Similarly, *Colletotrichum gloeosporioides s.s.* TS-09R upregulates some detoxification-related genes to resist the biocontrol of *B. amyloliquefaciens* [65]. In contrast, the genes encoding cell surface Cu-only superoxide dismutase (*ARB_03674*) and peperoxisomal catalase (*CAT1*) were downregulated 2.90-fold and 1.33-fold, respectively, suggesting that SFB-1 might disrupt detoxification and antioxidant homeostasis of *C. fimbriata*.

## 5. Conclusions

The results of this study showed that *B. amyloliquefaciens* strain SFB-1 significantly inhibits the mycelial growth of *C. fimbriata*, inducing mycelial swelling and suppressing spore germination, while also reducing lesion development in infected sweetpotato roots. Furthermore, the CFS of strain SFB-1 exhibited inhibitory effects on *C. fimbriata* growth *in vitro* assays. Transcriptome analysis revealed that SFB-1 affects key biological processes in *C. fimbriata*, including the downregulation of genes associated with cell wall integrity, membrane stability, and spore formation, suggesting that SFB-1 disrupts these critical physiological functions. Conversely, detoxification and antioxidant-related genes were upregulated, indicating that *C. fimbriata* may activate stress response mechanisms in response to SFB-1 treatment. These findings suggest that SFB-1 may be a potential biocontrol agent for the prevention and control sweetpotato black rot during postharvest storage. In future studies, we will aim to identify the antifungal substances in the CFS of SFB-1 that contribute to its inhibitory effects on *C. fimbriata*.

## Data Availability

The original contributions presented in the study are included in the article/Supplementary Material, further inquiries can be directed to the corresponding authors.

## Supplementary Materials

The following supporting information can be downloaded at: https://www.mdpi.com/article/10.3390/genes15121540/s1, Table S1: qRT-PCR primer sequences used in this study. Table S2: Statistical data on the sequencing results obtained for the transcriptome of *C. fimbriata*; Table S3: Details of enriched GO terms of genes; Table S4:Details of enriched KEGG pathway of genes. (a) Details of enriched KEGG pathway of up-regulated genes, (b) Details of enriched KEGG pathway of down-regulated genes.

## References

[1] Alam M.K. (2021). A Comprehensive Review of Sweet Potato (*Ipomoea batatas* [L.] Lam): Revisiting the Associated Health Benefits. Trends Food Sci. Technol..

[2] Parada-Rojas C.H., Pecota K., Almeyda C., Yencho G.C., Quesada-Ocampo L.M. (2021). Sweetpotato Root Development Influences Susceptibility to Black Rot Caused by the Fungal Pathogen *Ceratocystis fimbriata*. Phytopathology.

[3] Liu M., Meng Q., Wang S., Yang K., Tian J. (2023). Research Progress on Postharvest Sweet Potato Spoilage Fungi *Ceratocystis fimbriata* and Control Measures. Food Biosci..

[4] Marincowitz S., Barnes I., De Beer Z.W., Wingfield M.J. (2020). Epitypification of *Ceratocystis fimbriata*. Fungal Syst. Evol..

[5] Mohsin S.M., Hasanuzzaman M., Parvin K., Morokuma M., Fujita M. (2021). Effect of Tebuconazole and Trifloxystrobin on *Ceratocystis fimbriata* to Control Black Rot of Sweet Potato: Processes of Reactive Oxygen Species Generation and Antioxidant Defense Responses. World J. Microbiol. Biotechnol..

[6] Wamalwa L.N., Cheseto X., Ouna E., Kaplan F., Maniania N.K., Machuka J., Torto B., Ghislain M. (2015). Toxic Ipomeamarone Accumulation in Healthy Parts of Sweetpotato (*Ipomoea batatas* L. Lam) Storage Roots upon Infection by *Rhizopus stolonifer*. J. Agric. Food Chem..

[7] Li X., Liu M., Huang T., Yang K., Zhou S., Li Y., Tian J. (2021). Antifungal Effect of Nerol via Transcriptome Analysis and Cell Growth Repression in Sweet Potato Spoilage Fungi *Ceratocystis fimbriata*. Postharvest Biol. Technol..

[8] Tian J., Pan C., Zhang M., Gan Y.Y., Pan S.Y., Liu M., Li Y.X., Zeng X.B. (2019). Induced cell death in *Ceratocystis fimbriata* by pro-apoptotic activity of a natural organic compound, perillaldehyde, through Ca^2+^ overload and accumulation of reactive oxygen species. Plant Pathol..

[9] Reyes-Jurado F., Navarro-Cruz A.R., Ochoa-Velasco C.E., Palou E., López-Malo A., Ávila-Sosa R. (2020). Essential Oils in Vapor Phase as Alternative Antimicrobials: A Review. Crit. Rev. Food Sci. Nutr..

[10] Scruggs A.C., Basaiah T., Adams M.L., Quesada-Ocampo L.M. (2017). Genetic Diversity, Fungicide Sensitivity, and Host Resistance to *Ceratocystis fimbriata* Infecting Sweetpotato in North Carolina. Plant Dis..

[11] Li X., Li B., Cai S., Zhang Y., Xu M., Zhang C., Yuan B., Xing K., Qin S. (2020). Identification of Rhizospheric Actinomycete *Streptomyces lavendulae* Sps-33 and the Inhibitory Effect of Its Volatile Organic Compounds against *Ceratocystis fimbriata* in Postharvest Sweet Potato (*Ipomoea batatas* (L.) Lam.). Microorganisms.

[12] Zhang D.S., Zhang Y.C., Qiao Q., Qin Y.H., Tian Y.T., Wang S., Zhang Z.C. (2012). Toxicity and Co-toxicity of Ten Fungicides to *Ceratocystis fimbriata*. Agrochemicals.

[13] Xing K., Li T.J., Liu Y.F., Zhang J., Zhang Y., Shen X.Q., Li X.Y., Miao X.M., Feng Z.Z., Peng X. (2018). Antifungal and Eliciting Properties of Chitosan against *Ceratocystis fimbriata* in Sweet Potato. Food Chem..

[14] Tariq M., Khan A., Asif M., Khan F., Ansari T., Shariq M., Siddiqui M.A. (2020). Biological Control: A Sustainable and Practical Approach for Plant Disease Management. Acta Agric. Scand. Sect. B Soil Plant Sci..

[15] Salazar B., Ortiz A., Keswani C., Minkina T., Mandzhieva S., Pratap Singh S., Rekadwad B., Borriss R., Jain A., Singh H.B. (2023). *Bacillus* spp. as Bio-factories for Antifungal Secondary Metabolites: Innovation Beyond Whole Organism Formulations. Microb. Ecol..

[16] Wang J., Wang J., Liu T., Li X., Gao J., Jiang Y., Chen C.Q. (2023). *Bacillus amyloliquefaciens* FG14 as a potential biocontrol strain against rusty root rot of *Panax ginseng*, and its impact on the rhizosphere microbial community. Biol. Control..

[17] Khedher S.B., Mejdoub-Trabelsi B., Tounsi S. (2021). Biological potential of *Bacillus subtilis* V26 for the control of Fusarium wilt and tuber dry rot on potato caused by *Fusarium* species and the promotion of plant growth. Biol. Control.

[18] Wang X., Xie S., Mu X., Guan B., Hu Y., Ni Y. (2023). Investigating the resistance responses to *Alternaria brassicicola* in ‘Korla’ fragrant pear fruit induced by a biocontrol strain *Bacillus subtilis* Y2. Postharvest Biol. Technol..

[19] Wang T., Wang X., Han M., Song X., Yang D., Wang S., Shi X. (2021). Enhanced spoVF operon increases host attachment and biocontrol ability of *Bacillus subtilis* for the management of *Ceratocystis fimbriata* in sweet potato. Biol. Control.

[20] Zhu H., Wu S., Tang S., Xu J., He Y., Ren Z., Liu E. (2023). Isolation, identification and characterization of biopotential cyclic lipopeptides from *Bacillus subtilis* strain JN005 and its antifungal activity against rice pathogen *Magnaporthe oryzae*. Biol. Control.

[21] Han S., Chen J., Zhao Y., Cai H., Guo C. (2021). *Bacillus subtilis* HSY21 can reduce soybean root rot and inhibit the expression of genes related to the pathogenicity of *Fusarium oxysporum*. Pestic. Biochem. Physiol..

[22] Song J., Ling L., Xu X., Jiang M., Guo L., Pang Q., Wang W., Zhao J., Wang X. (2023). Biological control of gray mold of tomato by *Bacillus altitudinis* B1-15. Biol. Control.

[23] Malik J., Moosa A., Zulfiqar F., Aslam M.N., Albalawi M.A., Almowallad S., Mahmood T., Alasmari A., Yong J.W. (2024). Biocontrol potential of lipopeptides produced by the novel *Bacillus altitudinis* strain TM22A against postharvest *Alternaria* rot of tomato. LWT.

[24] Li B., Wang B., Pan P., Li P., Qi Z., Zhang Q., Lin R. (2019). *Bacillus altitudinis* strain AMCC 101304: A novel potential biocontrol agent for potato common scab. Biocontrol Sci. Technol..

[25] Jin X.B., Sun R., Zhu J., Xu Z., Liu Z., Wang Q., Ye X. (2012). Isolation and identification of *Bacillus altitudinis* ZJ 186 from marine soil samples and its antifungal activity against *Magnaporthe oryzae*. Curr. Res. Bacteriol..

[26] Siva M., Sreeja S.J., Thara S.S., Heera G., Anith K.N. (2023). Endophytic *Bacillus* spp. suppress *Rhizoctonia solani* web blight of bush cowpea. Rhizosphere.

[27] Wang B.J., Pu Q., Zhang Y.H., Zhang C., Wu H.T., Zeng G.H., Hu X.F. (2023). Secretion and volatile components contribute to the antagonism of *Bacillus velezensis* 1–10 against fungal pathogens. Biol. Control.

[28] Russi A., Granada C.E., Schwambach J. (2024). Optimization of *Bacillus velezensis* S26 sporulation for enhanced biocontrol of gray mold and anthracnose in postharvest strawberries. Postharvest Biol. Technol..

[29] Yousfi S., Krier F., Deracinois B., Steels S., Coutte F., Frikha-Gargouri O. (2024). Characterization of *Bacillus velezensis* 32a metabolites and their synergistic bioactivity against crown gall disease. Microbiol. Res..

[30] Zhang D., Huang K., Ye C., Zou D., Liu D., Wei X. (2024). Enhancing biological control of apple rot: Unveiling the antifungal potential and mechanism of *Bacillus amyloliquefaciens* HZ-12’s lipopeptide. Sci. Hortic..

[31] Kuang A., Fu X., Liu Z., Chen Q., Mao H. (2023). Biocontrol effect of the complex inoculants of Trichoderma and *Bacillus amyloliquefaciens* on chrysanthemum white rust. Biocatal. Agric. Biotechnol..

[32] Marković S., Milovanović T.P., Jelušić A., Iličić R., Medić O., Berić T., Stanković S. (2023). Biological control of major pathogenic bacteria of potato by *Bacillus amyloliquefaciens* strains SS-12.6 and SS-38.4. Biol. Control.

[33] Calvo H., Mendiara I., Arias E., Blanco D., Venturini M.E. (2019). The role of iturin A from *Bacillus amyloliquefaciens* BUZ-14 in the inhibition of the most common postharvest fruit rots. Food Microbiol..

[34] Ma T., Yang C., Cai F., Cui L., Wang Y. (2022). Optimizing fermentation of *Bacillus amyloliquefaciens* 3-5 and determining disease suppression and growth in cucumber (*Cucumis sativus*). Biol. Control.

[35] Liu F., Gao R., Zhang F., Ren Y., Li W., He B. (2023). Postharvest Biocontrol of Green Mold (*Penicillium digitatum*) in Citrus by *Bacillus velezensis* Strain S161 and Its Mode of Action. Biol. Control.

[36] Yang D., Sun H., Zhang C., Xu Z., Zhao Y., Xie Y. (2018). Biological Control of *Bacillus amyloliquefaciens* Strain XZ-1 Against Black Rot on Sweetpotato. Southwest China J. Agric. Sci..

[37] Wang C.J., Wang Y.Z., Chu Z.H., Wang P.S., Liu B.Y., Li B.Y., Yu X.L., Luan B.H. (2020). Endophytic *Bacillus amyloliquefaciens* YTB1407 Elicits Resistance Against Two Fungal Pathogens in Sweetpotato (*Ipomoea batatas* (L.) Lam.). J. Plant Physiol..

[38] Liu X., Bao T., Zheng L., Kgosi V.T., Liu X., Liu H. (2021). Cell Wall Integrity in *Magnaporthe oryzae* Is Weakened by Proteins Secreted by *Bacillus licheniformis* BL06. Biol. Control.

[39] Yousefvand M., Harighi B., Azizi A. (2023). Volatile Compounds Produced by Endophytic Bacteria Adversely Affect the Virulence Traits of *Ralstonia solanacearum*. Biol. Control.

[40] Sun M., Liu J., Li J., Huang Y. (2022). Endophytic Bacterium *Serratia plymuthica* from Chinese Leek Suppressed Apple Ring Rot on Postharvest Apple Fruit. Front. Microbiol..

[41] Gong Y., Liu J.Q., Xu M.J., Zhang C.M., Gao J., Li C.G., Ng K., Qin S. (2022). Antifungal Volatile Organic Compounds from *Streptomyces setonii* WY228 Control Black Spot Disease of Sweet Potato. Appl. Environ. Microbiol..

[42] Yang B.Y., Yang J.X., Wang G., Dong W.P., Xu P.L., Zheng Y., Yang W., Yao X.F., Xu J.H., Guo J.H. (2024). Tacrolimus analogue produced by *Bacillus amyloliquefaciens* HSSN09 suppresses watermelon Fusarium wilt by antagonizing FON. Biol. Control.

[43] Soulié M.C., Piffeteau A., Choquer M., Boccara M., Vidal-Cros A. (2003). Disruption of *Botrytis cinerea* class I chitin synthase gene Bcchs1 results in cell wall weakening and reduced virulence. Fungal Genet. Biol..

[44] Harayama T., Riezman H. (2018). Understanding the Diversity of Membrane Lipid Composition. Nat. Rev. Mol. Cell Biol..

[45] Choy H.L., Gaylord E.A., Doering T.L. (2023). Ergosterol Distribution Controls Surface Structure Formation and Fungal Pathogenicity. mBio.

[46] Jiang Y.Q., Lin J.P. (2022). Recent Progress in Strategies for Steroid Production in Yeasts. World J. Microbiol. Biotechnol..

[47] Yang J., Li C.F., Zhang Y.S. (2021). Engineering of *Saccharomyces cerevisiae* for 24-Methylene-Cholesterol Production. Biomolecules.

[48] Sun Z.J., Lian J.Z., Zhu L., Jiang Y.Q., Li G.S., Xue H.L., Wu M.B., Yang L.R., Lin J.P. (2021). Combined Biosynthetic Pathway Engineering and Storage Pool Expansion for High-Level Production of Ergosterol in Industrial *Saccharomyces cerevisiae*. Front. Bioeng. Biotechnol..

[49] Long N., Xu X., Zeng Q., Sang H., Lu L. (2017). Erg4A and Erg4B Are Required for Conidiation and Azole Resistance via Regulation of Ergosterol Biosynthesis in *Aspergillus fumigatus*. Appl. Environ. Microbiol..

[50] Gutiérrez M.S., Campusano S., González A.M., Gómez M., Barahona S., Sepúlveda D., Espenshade P.J., Fernández-Lobato M., Baeza M., Cifuentes V. (2019). Sterol Regulatory Element-Binding Protein (Sre1) Promotes the Synthesis of Carotenoids and Sterols in *Xanthophyllomyces dendrorhous*. Front. Microbiol..

[51] Stucky D.F., Arpin J.C., Schrick K. (2015). Functional Diversification of Two UGT80 Enzymes Required for Steryl Glucoside Synthesis in *Arabidopsis*. J. Exp. Bot..

[52] Zhang Y., Li T., Xu M., Guo J., Zhang C., Feng Z., Peng X., Li Z., Xing K., Qin S. (2021). Antifungal Effect of Volatile Organic Compounds Produced by *Pseudomonas chlororaphis* Subsp. *aureofaciens* SPS-41 on Oxidative Stress and Mitochondrial Dysfunction of *Ceratocystis fimbriata*. Pestic. Biochem. Physiol..

[53] Dijksterhuis J. (2019). Fungal Spores: Highly Variable and Stress-Resistant Vehicles for Distribution and Spoilage. Food Microbiol..

[54] Wu H., Tong Y., Zhou R., Wang Y., Wang Z., Ding T., Huang B. (2021). Mr-AbaA Regulates Conidiation by Interacting with the Promoter Regions of Both *Mr-veA* and *Mr-wetA* in *Metarhizium robertsii*. Microbiol. Spectr..

[55] Park H.S., Nam T.Y., Han K.H., Kim S.C., Yu J.H. (2014). VelC Positively Controls Sexual Development in *Aspergillus nidulans*. PLoS ONE.

[56] Höfer A.M., Harting R., Aßmann N.F., Gerke J., Schmitt K., Starke J., Bayram Ö., Tran V.T., Valerius O., Braus-Stromeyer S.A. (2021). The Velvet Protein Vel1 Controls Initial Plant Root Colonization and Conidia Formation for Xylem Distribution in *Verticillium wilt*. PLoS Genet..

[57] Togashi N., Yamashita A., Sato M., Yamamoto M. (2014). Functional Significance of Nuclear Export and mRNA Binding of Meiotic Regulator Spo5 in Fission Yeast. BMC Microbiol..

[58] Sun P., Zhang L., Li Z. (2022). Comparative Transcriptome Analysis Unravels the Response Mechanisms of *Cytospora mali* QH2 to a Biocontrol Agent, *Bacillus velezensis* L-1. Eur. J. Plant Pathol..

[59] Peng Y., Li S.J., Yan J., Tang Y., Cheng J.P., Gao A.J., Yao X., Ruan J.J., Xu B.L. (2021). Research Progress on Phytopathogenic Fungi and Their Role as Biocontrol Agents. Front. Microbiol..

[60] Liu R., Li J., Zhang F., Zheng D., Chang Y., Xu L., Huang L. (2021). Biocontrol Activity of *Bacillus velezensis* D4 Against Apple Valsa Canker. Biol. Control.

[61] Hirata A., Shimoda C. (1994). Structural Modification of Spindle Pole Bodies During Meiosis II Is Essential for the Normal Formation of Ascospores in *Schizosaccharomyces pombe*: Ultrastructural Analysis of spo Mutants. Yeast.

[62] Lu J., Holmgren A. (2014). The Thioredoxin Antioxidant System. Free Radic. Biol. Med..

[63] Gyaneshwari U., Swati K., Singh A.K., Prakash A., Pal P., Kumari B., Pandey B. (2023). Alcohol Dehydrogenase: Structural and Functional Diversity. Integrative Approaches to Biotechnology.

[64] Vo T.T., Jeong C.H., Lee S., Kim K.W., Ha E., Seo J.H. (2018). Versatility of ARD1/NAA10-Mediated Protein Lysine Acetylation. Exp. Mol. Med..

[65] Wang Q.H., Ji Y.P., Qu Y.Y., Qi Y.K., Li D.W., Liu Z.Y., Wu X.Q. (2020). The Response Strategies of *Colletotrichum gloeosporioides* ss Due to the Stress Caused by Biological Control Agent *Bacillus amyloliquefaciens* Deciphered by Transcriptome Analyses. Biol. Control.

